# Participant Preferences for the Provision of Registration Trials Results

**DOI:** 10.4021/jocmr1494w

**Published:** 2013-08-05

**Authors:** Soichiro Tajima, Akiyo Akaishi, Toshiko Miyamoto, Rumi Katashima, Kenichi Nakai, Takao Mitsui, Hiroaki Yanagawa

**Affiliations:** a Clinical Trial Center for Developmental Therapeutics, Tokushima University Hospital, Japan; b Department of Clinical Research, Tokushima National Hospital, National Hospital Organization, Japan

**Keywords:** Registration trials, Results, Disclosure, Participants, Preference

## Abstract

**Background:**

Clinical trials leading to drug approval (registration trials) play a central role in the drug development process, and attention has recently been paid to providing trial results to participants. In the present study, we examined the preferences of participants of registration trials for the provision of trial-related information.

**Methods:**

We used questionnaires to survey the preferences of registration trial participants at Tokushima University Hospital and Tokushima National Hospital. Of the 15 questions, 6 related to participant characteristics and the trials in which they participated, while 9 questions were concerned with preferences for the provision of information. A five-point scale (strongly agree, agree, neutral, disagree, and strongly disagree) was used, and positive answers (strongly agree and agree) were considered to indicate a positive preference.

**Results:**

Of the 58 subjects, 1 declined, giving a response rate of 98%. More than 70% of participants preferred to obtain information, even if they had served as controls. More than 80% of participants agreed to obtain information relating to trial results, even if the results were negative, and more than 80% of participants agreed to obtain information on the labeling state of the agent, even if development had ceased. Although more than 60% of participants agreed for the provision of information on their allocation and around more than 70% agreed to the provision of information on registration trials status, significantly fewer participants with difficult-to-treat diseases (for example, neurological and malignant diseases) agreed to obtain information compared with participants with other types of diseases (for example, acute, chronic, and psychological diseases). More than 50% of participants desired information to be provided directly by the physician, while a considerable number of participants desired information by means of clinical research coordinators (CRCs) (24.4%) or by posted letter (33.3%).

**Conclusion:**

The present results suggest the preferences for the provision of individual and overall information concerning research results. However, further study is warranted to determine participant preferences more precisely and the effect of the CRC-initiated infrastructure for providing information on patient satisfaction and for promoting registration trials.

## Introduction

Clinical trials leading to drug approval (registration trials) play a central role in the drug development process. To ethical conduct of clinical trials, various issues should be considered. Among these, recent attention has been paid to providing the results of registration trials to the participants [1]. Communication between investigators and participants seems to be important to promote registration trials, and in Japan, provision of registration trials results is mentioned in the plans for the promotion of registration trials by the Ministry of Health, Labor and Welfare and by the Ministry of Culture and Science of Japan.

The contribution of clinical research coordinators (CRCs) to registration trials is now widely recognized in Japan, including at Tokushima University Hospital, a University Hospital in a rural area [2]. At present, CRCs in contact with investigators provide trials results to participants on a demand basis. To establish a more systematic means of providing results, we examined participant preferences for receiving the results of registration trials at Tokushima University Hospital in collaboration with Tokushima National Hospital. Herein we present the results of the analysis.

## Methods

We assessed participant preferences for receiving the results of registration trials by conducting a questionnaire survey at Tokushima University Hospital and Tokushima National Hospital in a rural area of Japan.

Potential participants were those who had participated in registration trials at Tokushima University Hospital or Tokushima National Hospital between September 2010 and March 2011. One month after trial participation, CRCs provided participants with the details of the present study after obtaining the consent of the investigators of each trial. Questionnaires were then administered to participants who had given informed consent.

An anonymous questionnaire consisting of 15 questions divided into two parts was developed. The first part contained 6 questions relating to participant characteristics and the registration trials in which they participated. The second part contained 9 questions regarding preferences for the provision of information. A five-point scale (strongly agree, agree, neutral, disagree, and strongly disagree) was used to measure preferences. All participants were adults.

We compared participant preferences according to disease, and variables were analyzed using the Chi-squared test. All P values were based on two-sided tests, and P < 0.05 was considered significant.

This study was approved by the Ethics Committee of Tokushima University Hospital (#1054) and that of Tokushima National Hospital (#22-9).

## Results

### Respondent characteristics

Consent to be included in the present study was obtained from the investigators of the assessed registration trials. Of the 58 registered participants, 1 withdrew, giving a response rate of 98%. Respondents (n = 57) consisted of 28 males (49.1%) and 29 females (50.9%) aged 20 - 29 years (n = 2, 3.5%), 30 - 39 years (n = 6, 10.5%), 40 - 49 years (n = 8, 14.0%), 50 - 59 years (n = 10, 17.5%), 60 - 69 years (n = 18, 31.6%), 70 - 79 years (n = 11, 19.3%), and > 80 years (n = 2, 3.5%). In the registration trials, respondents were given agents against acute diseases (n = 3, 5.3%), chronic diseases (n = 15, 26.3%), neurological diseases (n = 29, 50.9%), malignant diseases (n = 7, 12.3%), and psychological diseases (n = 3, 5.3%).

### Respondent perception of the registration trials

To examine the perception of the registration trials, we asked participants to comment on the trial phase, duration, and placebo use. For the trial phase, 10 (17.5%) respondents answered phase II, 31 (54.4%) answered phase III, and the remaining 16 (28.1%) provided no answer. Duration was reported as less than 3 months by 2 (3.5%) respondents, 3 - 6 months by 7 (12.3%), 6 - 12 months by 8 (14.0%), 1 - 2 years by 14 (24.6%), more than 1 year by 20 (35.1%), and until registration by 1 (1.8%), while 5 (8.8%) provided no answer. Regarding placebo use, 33 (57.9%) respondents answered the trials were with placebo, 13 (22.8%) answered the trials were without placebo, and 11 (19.3%) provided no answer.

### Preferences for receiving allocation information

We asked participants about their preferences for receiving information on their own allocation, even if they had received placebo instead of the active drug. Because only some respondents had participated in registration trials that included placebo, participants of registration trials without placebo were encouraged to provide answers on the assumption that they had participated in registration trials that included placebo.

As shown in Table 1, more than 70% of respondents agreed (strongly agree and agree) to obtain information on their allocation, even if they received the placebo.

**Table 1:** Preference for Receiving Information on Allocation in a Registration Trial

	Strongly agree	Agree	Neutral	Disagree	Strongly disagree	No answer	Total
a. Do you want to be informed concerning your own allocation in the registration trial?	29 (50.9%)	14 (24.6%)	1 (1.8%)	3 (5.3%)	0	10 (17.5%)	57
b. Do you want to be informed concerning your own allocation in the registration trial, even if you were treated with placebo?	28 (49.1%)	14 (24.6%)	2 (3.5%)	2 (3.5%)	1 (1.8%)	10 (17.5%)	57

### Preferences for receiving information on registration trials results

As shown in Table 2, more than 80% of participants agreed (strongly agree and agree) to obtain information on trial results, even if the results were negative, and more than 80% of participants agreed (strongly agree and agree) to obtain information on the labeling state of the agent, even if development had ceased.

**Table 2:** Preference for Receiving Information on the Results of Registration Trials

	Strongly agree	Agree	Neutral	Disagree	Strongly disagree	No answer	Total
a. Do you want to be informed of the results (efficacy and safety) of the registration trial in which you participated?	34 (59.6%)	17 (29.8%)	2 (3.5%)	1 (1.8%)	1 (1.8%)	2 (3.5%)	57
b. Do you want to be informed of the results (efficacy and safety) of the registration trial in which you participated, even if negative?	31 (49.1%)	17 (29.8%)	4 (7.0%)	1 (1.8%)	1 (1.8%)	3 (5.3%)	57
c. Do you want to be informed of the state of labeling of the drug examined in the registration trial in which you participated?	32 (56.1%)	13 (22.8%)	5 (8.8%)	0	0	7 (12.3%)	57
d. Do you want to be informed of the state of labeling of the drug examined in the registration trial in which you participated, even if drug development has ceased?	30 (52.6%)	15 (26.3%)	7 (12.3%)	1 (1.8%)	0	4 (7.0%)	57

### Preferences for receiving information according to disease status

To examine the possibility that participant preferences differed according to the underlying disease, we categorized neurological diseases and malignant diseases as difficult-to-treat diseases and other types of diseases, which included acute diseases, chronic diseases, and psychological diseases.

As shown in Table 3, significantly fewer participants with difficult-to-treat diseases agreed to receive information on their allocation compared with those with other types of diseases, although more than 60% agreed to such provision.

**Table 3:** Preferences for Receiving Information on Allocation in the Trial and on the State of Registration Trials, Depending on the Type of Disease

	Participants who agreed to provision of information	
	Participants with difficult-to-treat diseases (n = 36)	Participants with other types of diseases (n = 21)
1-a. Do you want to be informed of your allocation in the registration trial?	23 (63.9%)	20 (95.2%) ^a^
1-b. Do you want to be informed of your own allocation in the registration trial, even if you were treated with placebo?	22 (61.1%)	20 (95.2%) ^b^
2-a. Do you want to be informed of the result (efficacy and safety) of the registration trial in which you participated?	30 (83.3%)	21 (100%) ^c^
2-b. Do you want to be informed of the result (efficacy and safety) of the registration trial that you participated, even if negative?	27 (75.0%)	21 (100%) ^d^
2-c. Do you want to be informed of the state of labeling of the drug examined in the registration trial in which you participated?	25 (69.4%)	20 (95.2%) ^e^
2-d. Do you want to be informed of the state of labeling of the drug examined in the registration trial in which you participated, even if drug development has ceased?	25 (69.4%)	20 (95.2%) ^f^

^a, b, c, d, e, f^Significant differences (P = 0.008, 0.005, 0.048, 0.013, 0.021, 0.021, respectively) compared with participants with difficult-to-treat diseases.

Regarding the status of the registration trials, although around 70% of respondents in both groups agreed to the provision, significantly fewer participants with difficult-to-treat diseases agreed to receive information compared with those with other types of diseases.

### Desired information

We asked participants to indicate desirable information by circling the corresponding options, and the following results were obtained: commercial name of labeled drug (n = 5), disease targeted by labeled drug (n = 7), price of labeled drug (n = 6), side effects of labeled drug (n = 15), and date of labeled drug authorization (n = 15).

In addition, we asked participants to indicate their preferences for receiving information on future registration trials involving agents against their disease if the trials were conducted in the same hospitals as past registration trials or in other hospitals in Tokushima Prefecture. Nine participants wanted information on trials in their hospital, while 1 wanted information involving other hospitals in Tokushima Prefecture.

### Preferences for means of receiving information

Preferences for means of receiving information are shown in Table 4. More than 50% of participants desired information direct from the physician in a face-to-face manner. A considerable number of participants desired information from CRCs (24.4%) or by posted letter (33.3%).

**Table 4:** Preferences Means of Receiving Information on the Results of Registration Trials

	Physician	CRC	Officer	No answer	Total
E-mail	1 (1.8%)	0	0	0	1 (1.8%)
Telephone	1 (1.8%)	1 (1.8%)	0	0	2 (3.5%)
Posted letter	8 (14.0%)	7 (12.3%)	4 (7.0%)	0	19 (33.3%)
Face-to-face	25 (43.9%)	6 (10.5%)	1 (1.8%)	0	32 (56.1%)
No answer	0	0	0	3 (5.3%)	3 (5.3%)
Total	35 (61.4%)	14 (24.6%)	5 (8.8%)	3 (5.3%)	57 (100%)

## Discussion

Although attention has been focused on the disclosure of study results to participants [1], investigators and CRCs in contact with investigators currently provide the results of trials to participants on a demand basis at Tokushima University Hospital and Tokushima National Hospital. To establish a more systematic means of providing results, we examined the preferences of participants for receiving the results of registration trials.

We examined participant attitudes on the provision of information regarding their allocation. Since not all participants had participated in registration trials, including trials with placebo, those who had participated in registration trials without placebo were encouraged to provide answers, on the assumption that they had participated in registration trials including placebo. As shown in Table 1, more than 70% of the participants preferred to receive information on their own allocation, even if they had received placebo instead of an active drug in the registration trial in which they had participated. In the present study, around 20% of the participants did not clearly express whether their registration trials included placebo. Joffe S at al [3] reported that misconceptions about cancer clinical trials are frequent among trial participants. Although there is a possibility that participants who had participated in registration trials without placebo could not realize the true meaning of placebo, the present finding suggests a positive preference for the provision of information on their allocation, and this tendency is consistent with that of previous reports involving study participants with chronic diseases [4-6].

Significantly fewer participants with difficult-to-treat diseases agreed to obtain information on their allocation compared with participants with other types of diseases, although more than 60% agreed to provision of such information (Table 3). These findings may indicate that more participants with difficult-to-treat diseases feared the uncertainty and potential disappointment caused by the provision, and this aspect should be considered in a future study.

Shalowitz and Miller [1] reviewed studies that reported a desire to receive results as a percentage of respondents; 90% (median; range 20 - 100%) of respondents wished to receive study results. In line with these findings, participants in the present study wanted to be informed of the results of the registration trial, even if negative or if drug development had ceased (Table 2). With regard to disease, significantly fewer participants with difficult-to-treat diseases (~70%) agreed to obtain the status of the registration trials compared with participants with other types of diseases (Table 3). Although the purpose of clinical trials, including registration trials, provides a societal benefit, many patients with no available therapy, such as some types of cancer patients, tend to participate in clinical trials, due potential personal benefits [7]. The reason for this difference is unclear at present, but one possible explanation is that participants with other types of diseases considered societal benefits as the reason for participating in the registration trials and were therefore more eager to know about the status of the registration trials. Further study is needed to precisely determine the preferences of patients with various diseases at various stages. Taken together, the present findings may show that feedback should be handled sensitively, depending on the disease.

Concerning the means of providing trial results to participants, investigations [8, 9] have shown posted letters to be preferable for adult participants. Brealey et al [10] reported that in participants in a pragmatic randomized trial, longer leaflets were preferred over shorter ones. In a chemotherapy trial for breast cancer, Johnson et al [11] reported that 40% of patients preferred to receive results via their hospital, while 47% preferred results posted directly to their home. In the present study, more participants wanted to receive their information in a face-to-face manner, while a considerable number of participants (33.3%) wanted the results delivered by post (Table 4). Considering that participants in the present study were treated regularly at the outpatient clinic, we regard our findings as being in line with those of previous reports.

In Japan, the contribution of CRCs in clinical trials has become well recognized. In a multicenter hypertension study, we found that physicians who could recruit participants into a trial considered the presence of a support system with CRCs as the reason to participate in the trial [12]. In the present study, a considerable number of trial participants (24.6%) had willingness to receive information from the CRC (Table 4). Practical issues, such as cost and time, were mentioned as barriers for investigators when informing participants of trial results [13, 14]. Establishing a CRC-initiated system for the provision of precise information may contribute to communication between investigators and participants, and moreover to investigator motivation.

The present study evaluated only the views of participants in a rural area of Japan. Nevertheless, we found that participants tended to prefer receiving information on the administered agent, even if placebo, and on the results of the registration trial, even if they had difficult-to-treat diseases. Buchwald H et al [15] reported that patients showed improved perceptions of their emotional quality of life, and moreover, patients became disposed to advise others to join a research study after disclosure of study results. Based on the present findings, we intend to establish a system for providing information. However, further study is warranted to determine the effect of the system on more effective communication between investigators and participants, as well as on the promotion of registration trials in rural areas.
